# Difference in basic concept of coronary bifurcation intervention between Korea and Japan. Insight from questionnaire in experts of Korean and Japanese bifurcation clubs

**DOI:** 10.1007/s12928-020-00742-7

**Published:** 2021-01-16

**Authors:** Yoshinobu Murasato, Yoshihisa Kinoshita, Junya Shite, Yutaka Hikichi, Chang-Wook Nam, Bon-Kwon Koo

**Affiliations:** 1 Department of Cardiology and Clinical Research Center, National Hospital Organization Kyushu Medical Center, 1-8-1, Jigyohama, Chuo, Fukuoka 810-8563 Japan; 2 Department of Cardiovascular Medicine, Toyohashi Heart Center, Toyohashi, Japan; 3 Department of Cardiology, Osaka Saiseikai Nakatsu Hospital, Osaka, Japan; 4 Department of Cardiology, Saga University, Saga, Japan; 5 Division of Cardiology, Keimyung University Dongsan Hospital, Daegu, South Korea; 6 Department of Internal Medicine and Cardiovascular Center, Seoul National University Hospital, Seoul, South Korea

**Keywords:** Bifurcation, Drug-eluting stent, Fractional flow reserve, Intravascular ultrasound, Optical coherence tomography

## Abstract

The coronary bifurcation intervention varies among countries due to the differences in assessment of lesion severity and treatment devices. We sought to clarify the difference in basic strategy between South Korea and Japan. A total of 19 and 32 experts from Korean (KBC) and Japanese Bifurcation Clubs (JBC), respectively, answered a survey questionnaire concerning their usual procedure of coronary bifurcation intervention. JBC experts performed less two-stent deployment in the left main (LM) bifurcation compared to KBC experts (JBC vs. KBC: median, 1–10% vs. 21–30%, *p* < 0.0001) instead of higher performance of side branch dilation after cross-over stenting in both LM (60% vs. 21%, *p* = 0.001) and non-LM bifurcations (30% vs. 5%, *p* = 0.037). KBC experts more frequently performed proximal optimization technique (POT) in non-LM bifurcation (41–60% vs. 81–99%, *p* = 0.028) and re-POT in both LM (1–20% vs. 81–99%, *p* = 0.017) and non-LM bifurcations (1–20% vs. 81–99%, *p* = 0.0003). JBC experts more frequently performed imaging-guided percutaneous coronary intervention, whereas KBC experts more often used a pressure wire to assess side branch ischemia. JBC experts used a rotablator more aggressively under the guidance of optical coherence tomography. We clarified the difference in the basic strategy of coronary bifurcation intervention between South Korea and Japan for better understanding the trend in each country.

## Introduction

Providing provisional treatment and reducing two-stent deployment for coronary bifurcation lesions has gained world-wide acceptance to avoid unnecessary treatment and reduce the risk of adverse cardiac events [1–3]. In addition, proximal optimization technique (POT) has been widely used instead of routine kissing balloon inflation (KBI) for the optimal dilation of proximal main vessels (MV) [1, 2]. However, interventions for coronary bifurcation remain challenging because large variations in lesion anatomy, method of assessment of lesion severity, and treatment devices make standardization of treatment difficult. The frequency of two-stent deployment, POT and KBI, penetration under imaging- or physiologic-guidance, and usage of atherectomy devices still widely vary depending on the operator’s skill and judgment, institution-specific practices, and social circumstance.

In Asia, South Korea and Japan lead in the number of coronary interventions performed annually, and both imaging- and physiologic-guidance are popular in these countries. However, these countries follow different practices for bifurcation treatment probably due to differences in concept or philosophy.

We sought to investigate the basic strategy of coronary bifurcation treatment using a questionnaire answered by experts in the Japanese Bifurcation Club (JBC) and Korean Bifurcation Club (KBC) and to clarify the differences between the practices followed in these countries.

## Methods

We prepared both paper and internet survey questionnaires for usual bifurcation treatment in left main (LM) and non-LM lesions with side branch (SB) diameter ≥ 2.5 mm. The questionnaire included the following items: background of percutaneous coronary intervention (PCI) operator and institute, two-stent deployment, POT/re-POT, method of SB dilation, status of usage of the devices, imaging-guidance, and rotablation (Table 1). Thirty-two and 19 experts in JBC and KBC answered the questionnaire, respectively.

**Table 1:** Questionnaire of the survey of usual coronary bifurcation treatment

Question		LM	Non-LM	Options						
Background										
1	How many PCI cases are performed in your institute annually?			a. ≤ 100	b. 101–300	c. 301–700	d. 701–1000	e. 1001–2000	f. ≥ 2001	
2	How many PCI cases do you perform annually?			a. ≤ 50	b. 51–100	c. 101–200	d. 201–300	e. ≥ 301		
3	How long is your career as a PCI operator?			a. ≤ 5 years	b. 5–10 years	c. 11–20 year	d. ≧ 21 years			
2-stent										
4	How often is the 2-stent deployment performed in the coronary bifurcation lesion in your institute?			a. 0%	b. 1–10%	c. 11–20%	d. 21–30%	e. 31–40%		
5	Please select a 2-stent technique that apply									
	Most frequently used technique			a. Culotte	b. Crush (classic, mini)	c. DK-crush	d. T-stenting	e. TAP		
	Secondly used technique			a. Culotte	b. Crush (classic, mini)	c. DK-crush	d. T-stenting	e. TAP		
Proximal optimization technique (POT)										
6	How often do you perform the POT after cross-over stenting in the bifurcation lesion with the size of side branch (SB) of more than 2.5 mm?			a. 0%	b. 1–20%	c. 21–40%	d. 41–60%	e. 61–80%	f. 81–99%	g. 100%
7	How often do you perform the re-POT after cross-over stenting followed by any kind of SB dilation in the bifurcation lesion with the SB size of more than 2.5 mm?			a. 0%	b. 1–20%	c. 21–40%	d. 41–60%	e. 61–80%	f. 81–99%	g. 100%
SB dilation										
8	How often do you perform the SB dilation after cross-over stenting in the bifurcation lesion with the SB size of more than 2.5 mm?			a. 0%	b. 1–20%	c. 21–40%	d. 41–60%	e. 61–80%	f. 81–99%	g. 100%
9	Please rank the order of your favourite SB dilation method			a. KBI	b. Modified KBI	c. POT + only SB dilation				
10	Please answer the number of the following devices that you use in case of your standard treatment of cross-over stenting followed by KBI or SB dilation									
	(1) balloon			a. 1	b. 2	c. 3	d. 4			
	(2) guide wire			a. 1	b. 2	c. 3	d. 4			
	(3) imaging device(IVUS/OCT/OFDI)			a. None	b. IVUS	c. OCT/OFDI	d. IVUS + OCT/OFDI			
	(4) pressure wire			a. None	b. Routinely or in case of necessary					
	(5) double lumen catheter (DLC)/micro catheter (MC)			a. Neither	b. DLC	c. MC	d. Both			
Imaging										
11	Please answer the frequency of the following investigation performed in your standard imaging-guided PCI									
	(1) Pre-PCI; MV pullback			a. 0%	b. 1–20%	c. 21–40%	d. 41–60%	e. 61–80%	f. 81–99%	g. 100%
	(2) Pre-PCI; SB pullback			a. 0%	b. 1–20%	c. 21–40%	d. 41–60%	e. 61–80%	f. 81–99%	g. 100%
	(3) After guide wire re-crossing; MV pullback			a. 0%	b. 1–20%	c. 21–40%	d. 41–60%	e. 61–80%	f. 81–99%	g. 100%
	(4) Post-PCI; MV pullback			a. 0%	b. 1–20%	c. 21–40%	d. 41–60%	e. 61–80%	f. 81–99%	g. 100%
	(5) Post-PCI; SB pullback			a. 0%	b. 1–20%	c. 21–40%	d. 41–60%	e. 61–80%	f. 81–99%	g. 100%
Rotablator										
12	How many percentage is the rotablator performed in the whole PCI cases?			a. 1–10%	b. 11–20%	c. 21–30%				
13	How often do you finish the rotablation procedure after using only one burr?			a. 0%	b. 1–20%	c. 21–40%	d. 41–60%	e. 61–80%	f. 81–99%	g. 100%
14	How often do you use the imaging guidance in the rotablation?			a. 0%	b. 1–20%	c. 21–40%	d. 41–60%	e. 61–80%	f. 81–99%	g. 100%
15	In case of imaging-guided rotablation			a. 0%	b. 1–20%	c. 21–40%	d. 41–60%	e. 61–80%	f. 81–99%	g. 100%
	(1) How often do you use the IVUS?									
	(2) How often do you use the OCT/OFDI?			a. 0%	b. 1–20%	c. 21–40%	d. 41–60%	e. 61–80%	f. 81–99%	g. 100%
16	How often do you use the rotablation for plaque modification in the SB?			a. 0%	b. 1–5%	c. 6–10%	d. 11–20%	e. 21–30%	f. > 30%	

*LM* left main, *PCI* percutaneous coronary intervention, *KBI* kissing balloon inflation, *IVUS* intravascular ultrasound, *OCT* optical coherence tomography, *OFDI* optical frequency domain imaging, *MV* main vessel

### Statistical analysis

Data were presented as median for discrete variables and ordinal scales. Discrete variables were compared between the groups using a Fisher’s exact test, while ordinal scales were compared using a Mann–Whitney *U* test. All *p* values were two-sided and considered statistically significant at levels < 0.05. All statistical analyses were performed with EZR software (Saitama Medical Center, Jichi Medical University, Saitama, Japan), a modified version of R commander (The R Foundation for Statistical Computing, Vienna, Austria).

## Results

### Background of PCI operator and institute

**Fig. 1:**
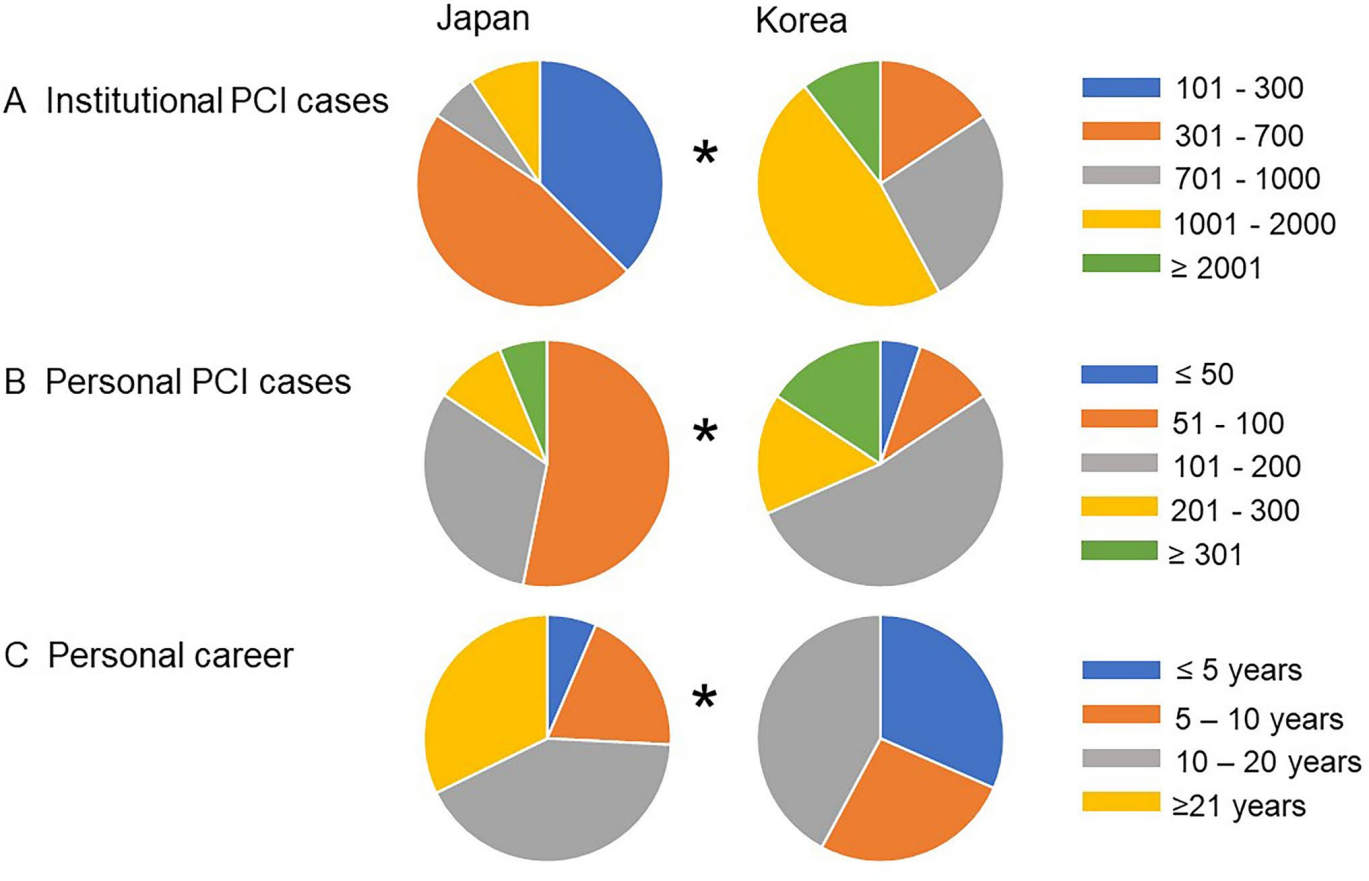
Comparison between experts of Japanese Bifurcation Club (JBC) and Korean Bifurcation Club (KBC) in terms of background. **a** Institutional annual PCI cases. **b** Personal annual PCI cases. **c** Personal career as a PCI operator. *p < 0.05 in comparison of JBC vs. KBC

JBC experts belonged to an institute wherein few PCI cases were handled compared to the institutes where the KBC experts were employed (median: JBC 301–700 cases/institute vs. KBC 1001–2000 cases/institute, *p* < 0.0001). The annual PCI cases handled by an expert were less in Japan, compared to South Korea (JBC 101–200 cases vs. KBC 201–300 cases, *p* = 0.022). Conversely, the experts from Japan had a longer tenure than the experts from South Korea (*p* = 0.002, ≥ 21 years; JBC 32% vs. KBC 0%) (Fig. 1).

### Two-stent deployment

**Fig. 2:**
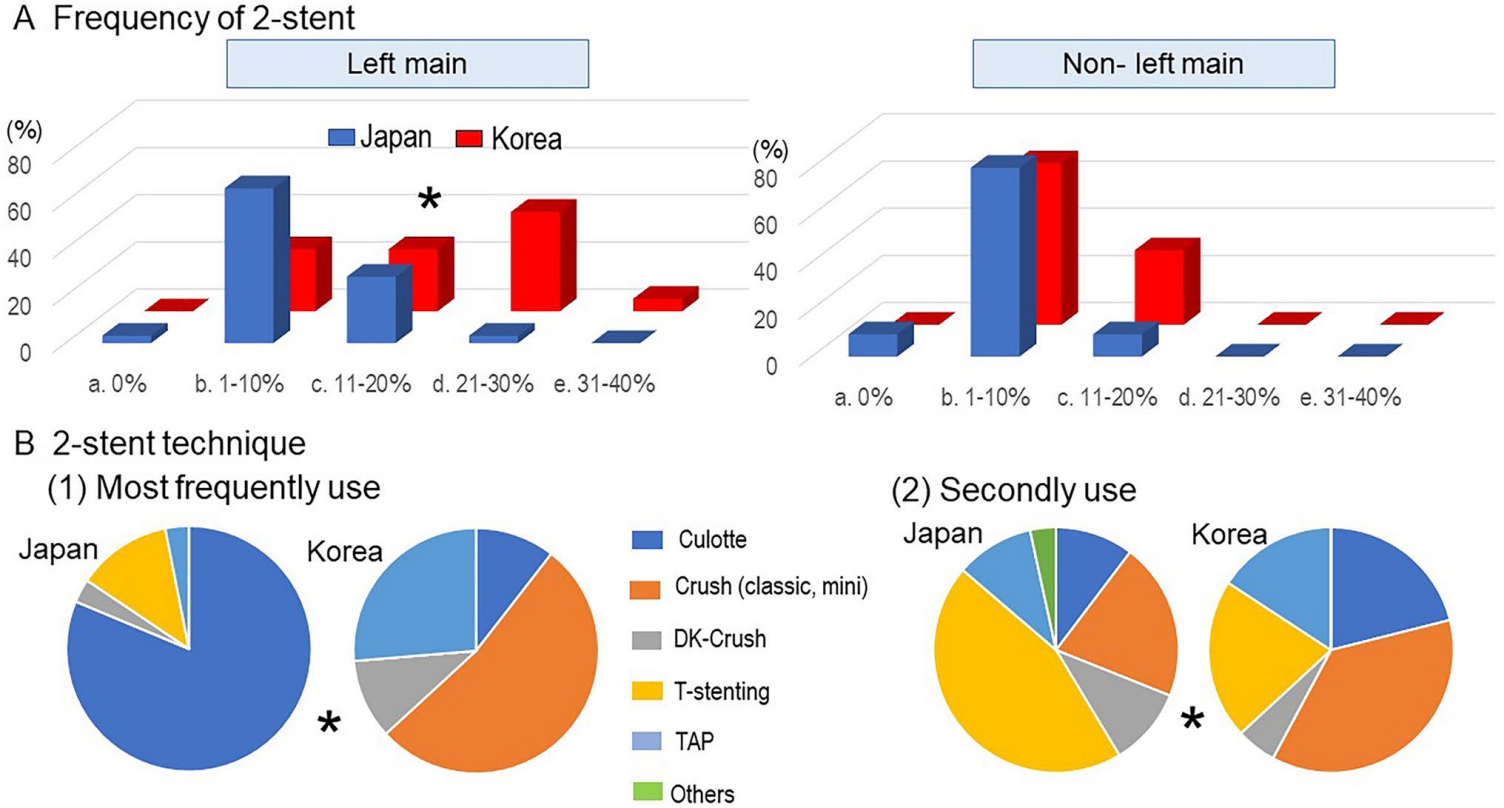
Comparison between JBC and KBC experts in 2-stent technique. **a** Frequency of 2-stent in left main (left) and non-left main bifurcations (right). **b** 2-stent technique. (1) Most frequently used technique. (2) Secondly used technique. *p < 0.05 in comparison of JBC vs. KBC

Two-stent deployment was performed more often for LM bifurcation among KBC experts (JBC 1–10% vs. KBC 21–30%, *p* < 0.0001), whereas the number was not significantly different between the groups for non-LM bifurcation (Fig. 2a). The most and second most frequently used two-stent techniques were completely different, which were culotte (81%) and T-stenting (45%) in JBC experts and crush stenting in both groups (53% and 37%) in KBC experts (*p* < 0.0001) (Fig. 2b).

### POT/re-POT

**Fig. 3:**
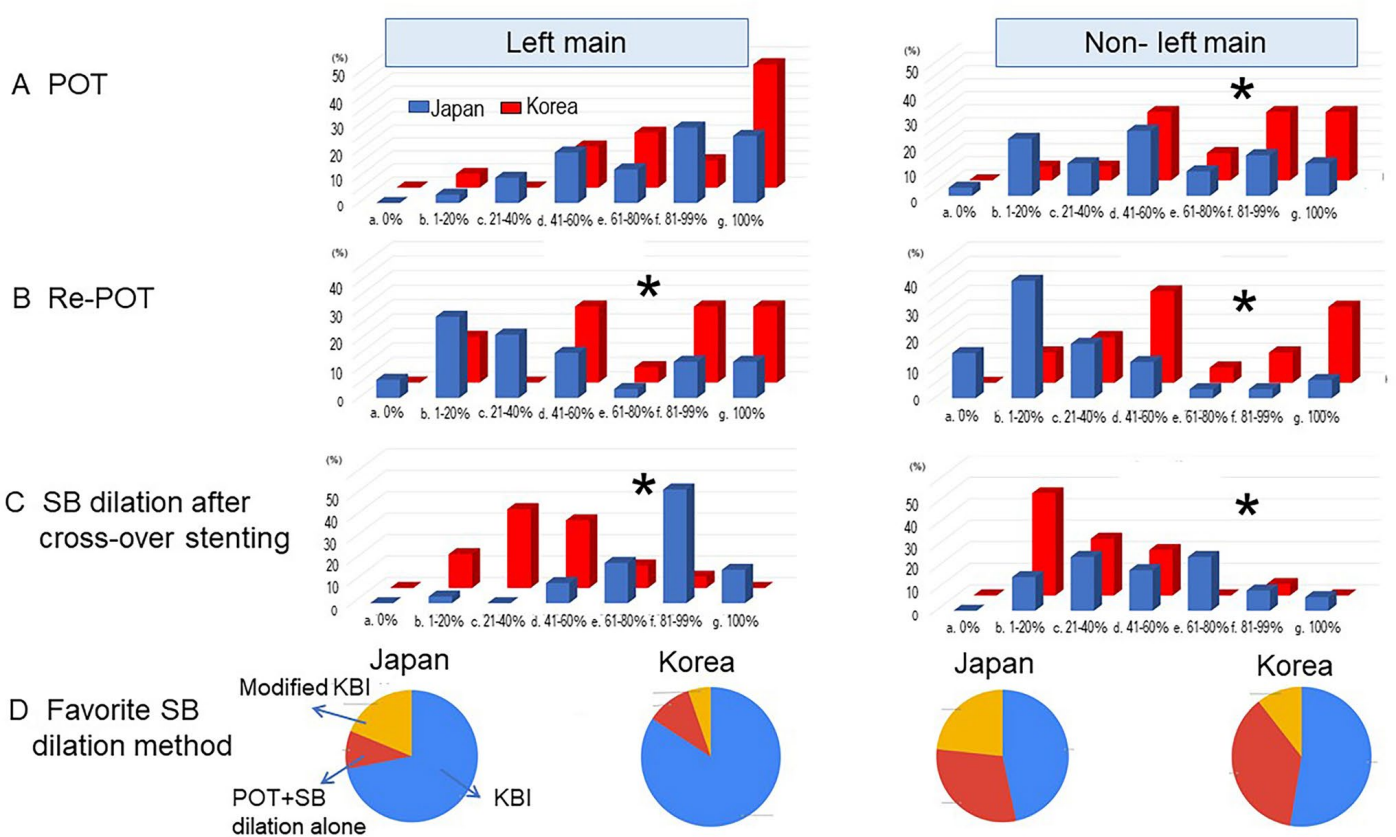
Comparison between JBC and KBC experts in proximal optimization technique (POT)/re-POT and side branch (SB) dilation in cross-over stenting. **a** Frequency of POT in left main (left) and non-left main bifurcations (right). **b** Frequency of re-POT. **c** Frequency of SB dilation. **d** Favourite method of SB dilation. *KBI* kissing balloon inflation. *p < 0.05 in comparison of JBC vs. KBC

Although routine performance of the POT in the LM bifurcation was numerically more frequent in KBC experts (JBC 26% vs. KBC 47%), there was no statistically significant difference. The POT performance in the non-LM bifurcation was more in KBC experts (JBC 41–60% vs. KBC 81–99%, *p* = 0.028) (Fig. 3a). In terms of the re-POT, KBC experts performed more frequently in both LM (JBC 1–20% vs. KBC 81–99%, *p* = 0.017) and non-LM bifurcations (JBC 1–20% vs. KBC 81–99%, *p* = 0.0003) (Fig. 3b).

### SB dilation

**Fig. 4:**
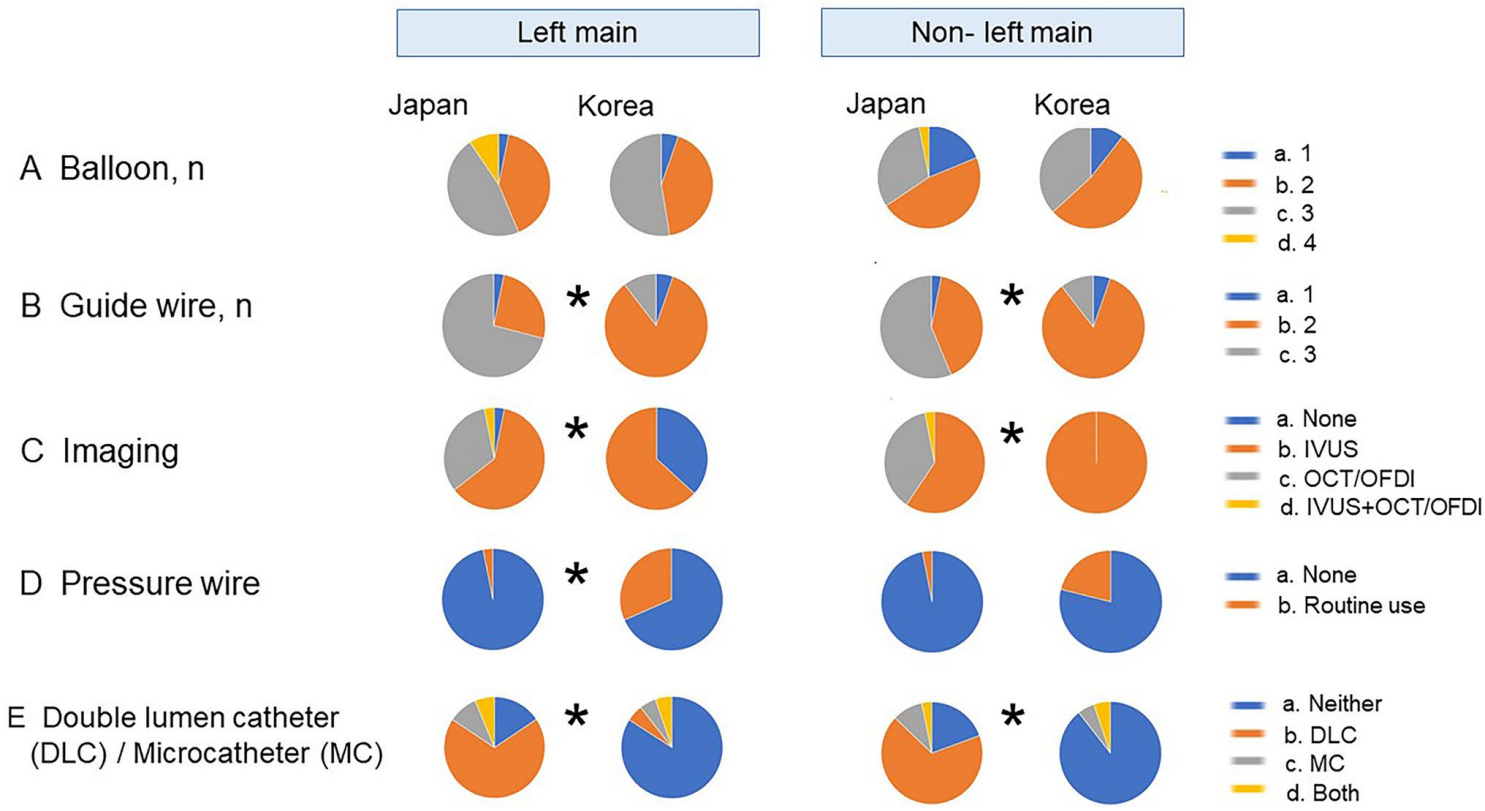
Comparison between JBC and KBC experts in routine use of the devices for usual cross-over stenting. **a** Number of balloons. **b** Number of guide wires. **c** Imaging device. *IVUS* intravascular ultrasound, *OCT* optical coherence tomography, *OFDI* optical frequency domain imaging. **d** Use of pressure wire. **e** Use of double lumen catheter (DLC) and/or microcatheter (MC). *p < 0.05 in comparison of JBC vs. KBC

The incidence of SB dilation after cross-over stenting was higher in JBC experts in both LM (JBC 81–99%, KBC 21–40%, *p* < 0.0001) and non-LM bifurcations (JBC 61–80%, KBC 1–20%, *p* = 0.004) (Fig. 3c). Kissing balloon inflation (KBI) was most frequently used in both LM and non-LM bifurcations in both groups of experts (Fig. 3d). In the case of a negative value of fractional flow reserve (FFR)/instantaneous wave-free ratio (iFR) in the stent-jailed SB, more JBC experts thought SB dilation with KBI or simple dilation was necessary in LM (JBC 60% vs. KBC 21%, *p* = 0.001) and non-LM bifurcations (JBC 30% vs. KBC 5%, *p* = 0.037), while KBC experts made the negative results of physiological assessment more important to reduce SB dilatation (Fig. 4).

### Device (balloon, guide wire, imaging device, pressure wire, and microcatheter/double lumen catheter)

In the LM bifurcation, JBC experts used more guide wires (JBC 3 vs. KBC 2, *p* < 0.0001) and double lumen catheters routinely (JBC 75% vs. KBC 11%, *p* < 0.0001), whereas routine use of pressure wires for the assessment of the jailed SB stenosis was less frequent (JBC 3% vs. KBC 32%, *p* = 0.008). In the non-LM bifurcation, a similar trend was found: JBC experts used more guide wires (JBC 3 vs. KBC 2, p = 0.002) and double lumen catheters (JBC 71% vs. KBC 5%, *p* < 0.0001), with less routine use of pressure wires (JBC 3% vs. KBC 21%, *p* = 0.06) (Fig. 4).

### Imaging guidance (device and timing)

**Fig. 5:**
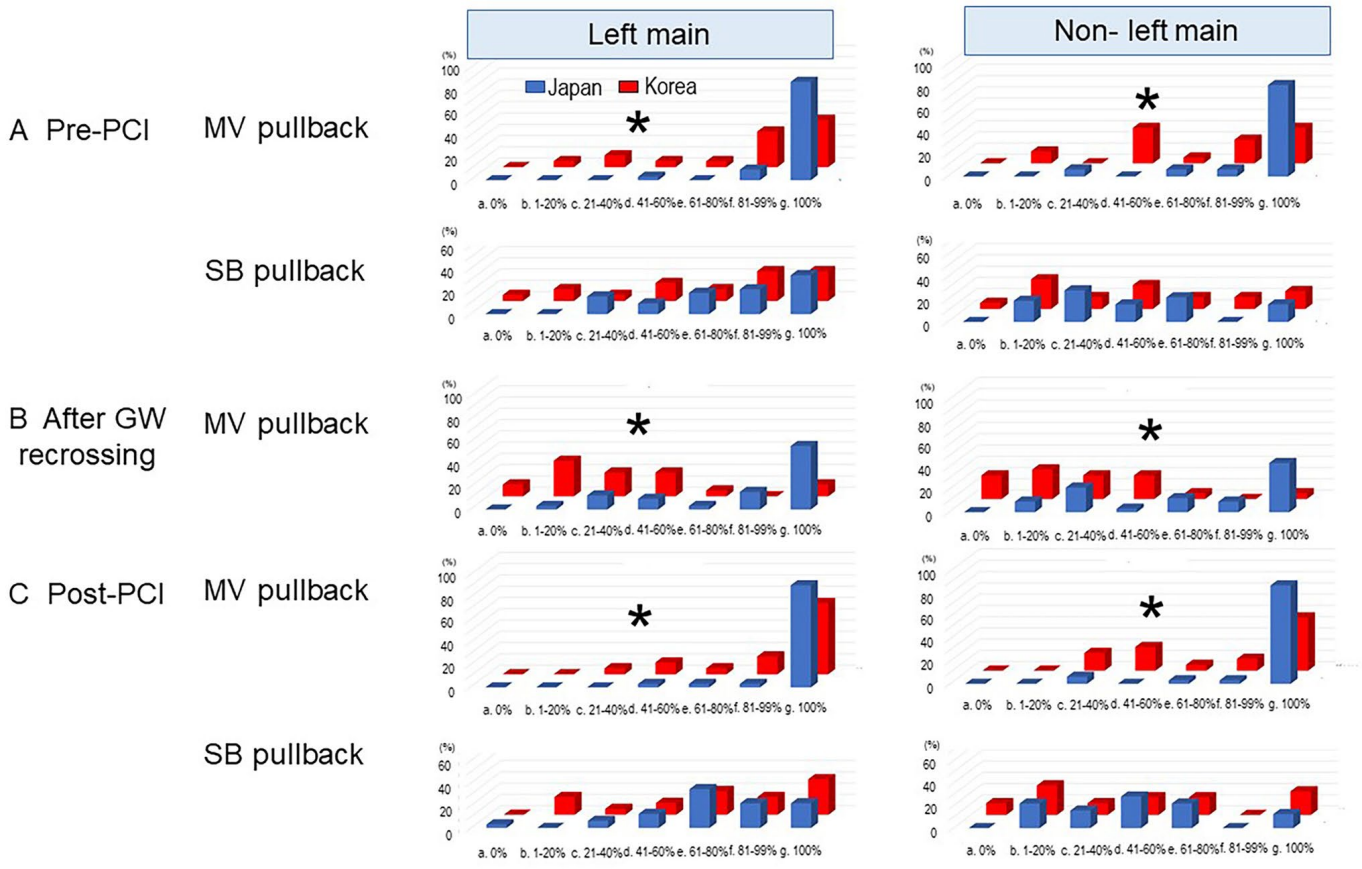
Comparison between JBC and KBC experts in frequency of the imaging observation in left main (left) and non-left main bifurcations (right). **a** Pre-PCI. Main vessel (MV, upper) and side branch (SB) pullbacks (lower). **b** After guide wire (GW) re-crossing. **c** Post-PCI. Main vessel (MV, upper) and side branch (SB) pullbacks (lower). *p < 0.05 in comparison of JBC vs. KBC

As for imaging device, JBC experts usually used intravascular ultrasound (IVUS) or optical coherence tomography (OCT)/optical frequency domain imaging (OFDI), which was more frequently in the LM bifurcation (JBC 94% vs. KBC 63%, *p* = 0.0004). JBC experts used OCT/OFDI in about 40% in both LM and non-LM bifurcations, while KBC experts never primarily used them. In the LM bifurcation, pullbacks from main vessels (MV) in both pre- (*p* = 0.0005) and post-PCI (*p* = 0.019) were popular in both groups of experts; however, JBC experts performed this technique more frequently. Pullbacks from SB in both pre- and post-PCI were performed less than those in MV, and frequencies were not significantly different between JBC and KBC experts. However, assessment of guide wire re-crossing point to the SB was more frequently performed in JBC experts (JBC 100% vs. KBC 1–20%, *p* < 0.0001). In the non-LM bifurcation, KBC experts perform less assessment of the MV in both pre- (*p* = 0.0005) and post-PCI (*p* = 0.003) as well as that of the guide wire re-crossing point (JBC 100% vs. KBC 1–20%, *p* = 0.0002) (Fig. 5).

### Rotablation (frequency, burr size, imaging-guidance, and SB treatment)

**Fig. 6:**
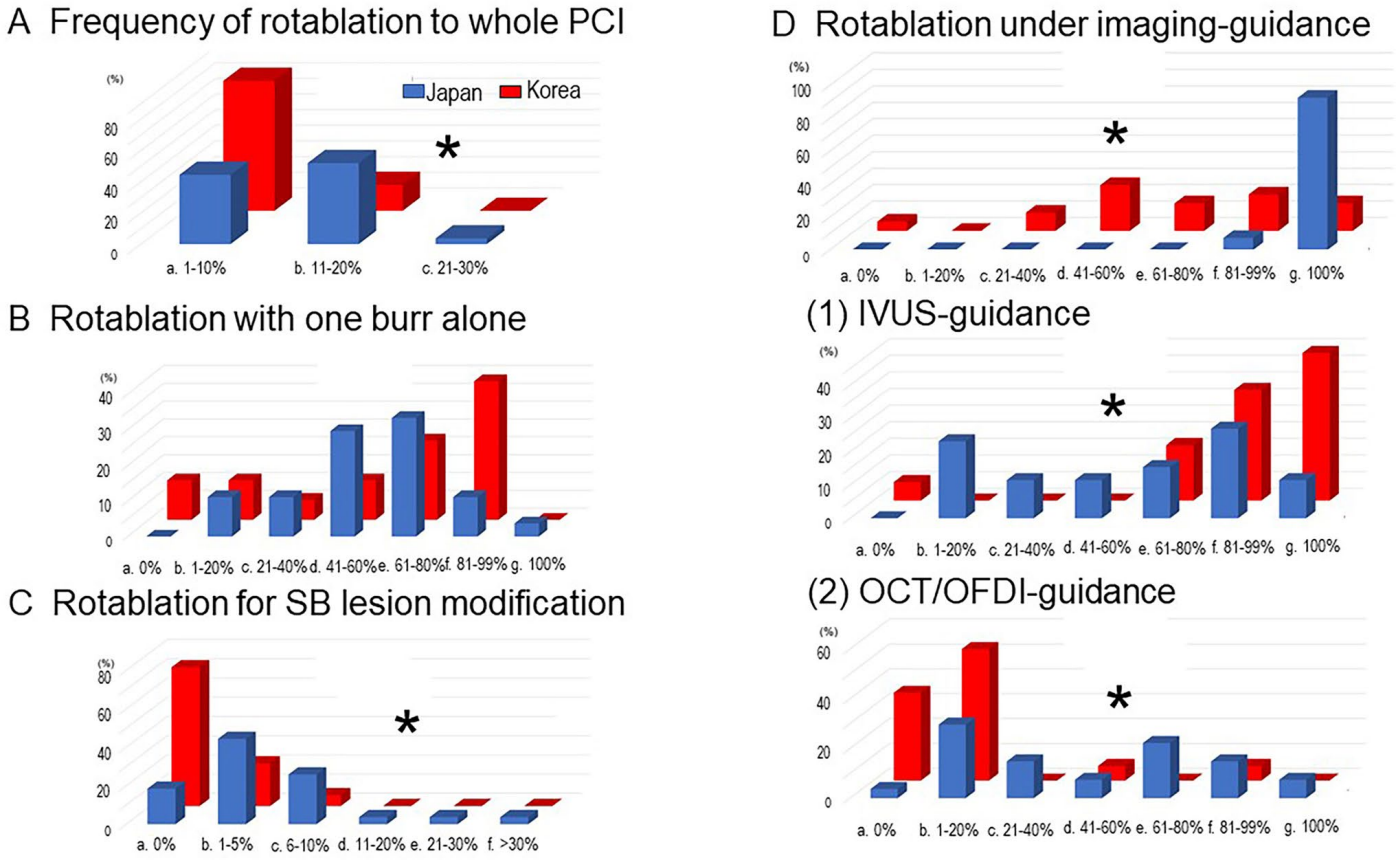
Comparison between JBC and KBC experts in rotablation. **a** Frequency of rotablation to whole PCI. **b** Frequency of rotablation with one burr alone. **c** Frequency of rotablation for SB lesion modification. **d** Rotablation under imaging-guidance. (1) Frequency of IVUS-guidance. (2) Frequency of OCT/OFDI-guidance. *p < 0.05 in comparison of JBC vs. KBC

Rotablation was more frequently used in JBC experts (JBC 11–20% vs. KBC 1–10%%, *p* = 0.01). JBC experts performed rotablation under the imaging guidance more frequently (JBC 100% vs. KBC 41–60%, *p* < 0.0001) with more OCT/OFDI guidance (*p* = 0.0003), while KBC experts mainly used the IVUS (*p* = 0.003). JBC experts used rotablation more aggressively for SB plaque modification (JBC 1–5% vs. KBC 0% %, *p* = 0.0003) (Fig. 6).

## Discussion

The present study clarified the difference in basic concept of coronary bifurcation treatment between JBC and KBC experts.

### Social background

There were more small-volume PCI centres and few operator’s personal annual cases among JBC experts; however, they had a longer operator’s career. Intensification to the large cardiovascular centre has been advanced in South Korea, which leads to more experience in younger operators. Physiological assessment using a pressure wire and imaging device during PCI are reimbursed by social insurance in Japan, whereas this is not done in South Korea. Penetration of imaging-guidance is more than 90% in daily practice in Japan [the annual report in 2014 of the Japanese Registry of All Cardiac and Vascular Disease (http://www.j-circ.or.jp/jittai_chosa/jittai_chosa2014web.pdf)], while 39% in Korean coronary bifurcation registry (COBIS) II [4]. The difference in imaging-guidance in daily practice may have some influence on basic concept.

### Two-stent deployment

Two-stent deployment is more frequently used by KBC experts, which is within the acceptable range in comparison with pivotal international studies [5–8], while JBC experts strictly limit two-stent deployment. Since frequency of true bifurcation lesion does not seem to differ between Japan and South Korea, the difference in frequency of two-stent deployment may mainly attribute to their response to pre-PCI SB lesion severity (stenosis and lesion length) and that to the condition after SB dilation (remained stenosis and dissection). Although KBC experts perform more physiological assessment in the SB and leave the SB without any balloon dilation more than JBC experts, more frequency of two-stenting in South Korea indicates more performance of provisional or planned two-stenting once they decide to treat the SB. Angiographically hazy image in the SB without imaging guidance is likely to induce more performance of two-stenting in Korea where 60% of bifurcation PCI is not performed under the imaging guidance, while mild dissection clearly detected by the imaging is likely to be left without stenting in Japan with higher penetration of the imaging guidance. In the DEFINITION trial, two-stenting brought better survival-free rate from adverse cardiac events compared to provisional stenting in the complex true bifurcation lesion [8]. Conversely, many randomized trials with comparison between provisional stenting and two-stenting demonstrated worse [9, 10] or ineffective outcomes after two-stenting [11, 12] except for DK-crush V study [13]. The proportion of 2-stent deployment has been decreased in both Japan (29.6% [14] to 21% [15]) and South Korea (40.3% [4] to 23.2% [16]), because more adverse cardiac events were observed in their initial experience of 1st-generation DES [14, 17]. Favorable clinical outcome of 2-stent with new-generation DES similarly as that of provisional stenting has been reported in Korean registry [17] but not in Japanese registry [15]. Japanese experts have continued more effort so as to leave more mildly dissected SB without stenting, to treat with drug-coated balloon and to ablate the SB plaque for the reduction of SB stenting. However, these attempts have not yet been investigated in a large-scale study and ideal proportion of 2-stent providing both clinical and cost benefits is still argued.

The primary stenting technique was culotte stenting in JBC experts and crush stenting in KBC experts. In Korean registries, crush stenting was most frequently used in 41–53% and culotte stenting was less used in 2–12% [4, 18]. On the contrary, there was higher prevalence of culotte stenting in Japan (16–81%) [14, 19]. After introducing new-generation DES, TLR was dramatically reduced in Korea (16.1–7.8%) [17] and Japan (13.6–6.7%) [14, 19]. Culotte stenting provides a certain DES coverage in the whole bifurcation with less layer; however, there are risks of MV or SB occlusion during the procedure as well as inadequate expansion of branch ostium due to jailed strut [20–22]. Crush stenting has a much lower risk of branch loss due to its protective procedure for MV occlusion and a certain SB stenting; however, there remain three-layered overlapping struts in the proximal MV and difficulty in SB opening [20–22]. In the DK-crush III study, randomized comparison was done between DK-crush and culotte stentings in unprotected LM bifurcation, and DK-crush stenting was superior in free survival from major adverse cardiac events and target lesion revascularization [23]. However, a meta-analysis comparing between culotte and crush stentings did not show superiority in either of them [24]. Since both 2-stenting required specific complicated procedures, performing with operator’s familiar technique may lead to more optimal procedure and better clinical outcome.

### POT/re-POT

POT can provide symmetric adequate stent expansion in the proximal MV with appropriate widening of the jailed struts at the SB ostium, which facilitates optimal guide wire re-crossing to the distal cell for the SB dilation [25, 26]. Both JBC and KBC experts realized the efficacy of the POT and performed it with higher frequency. Re-POT is recommended to correct the stent deformation or malapposition induced by KBI or SB dilation [25]; however, a recent study demonstrated risks of worsening SB obstruction [27] and stent deformation by insertion of the re-wrapped POT balloon. KBC experts performed more re-POT, while JBC experts does not aggressively. JBC experts generally considered that re-POT is not mandatory, when adequate stent expansion without any significant malapposition or stent deformation is confirmed in the imaging [28, 29]. Re-POT is an essential procedure in angio-guided bifurcation PCI to secure adequate stent expansion without malapposition.

### SB dilation according to physiological assessment versus imaging guidance

JBC experts performed SB dilation after cross-over stenting more aggressively in both LM and non-LM bifurcation lesions with large SB (≥ 2.5 mm) as shown in Fig. 3c, while KBC experts assessed more FFR/iFR and treated the SB more strictly according to its results as shown in Fig. 4d. FFR measurement in the jailed SB revealed that 70% of angiographically significant stenosis did not present a significant FFR value (< 0.80) [30], and FFR-guided deferring SB treatment did not show worsening of adverse cardiac events at long-term follow-up [31, 32]. Korean experts are more unlikely to perform SB dilation according to their experience of physiology-guided deferring of the jailed SB treatment [30–32]. However, in the bench testing and computer simulation, jailed struts at the SB ostium introduces flow turbulence and generates a low shear stress area [33], which is likely to provoke atherosclerotic change. The OCT observation of the jailed SB without KBI treatment at 9–12-month follow-up revealed more thrombus attachment compared to the opened SB with KBI [34]. A greater number of jailed struts in the SB ostium was associated with SB luminal narrowing at follow-up [35]. JBC experts used more OCT/OFDI and confirmed the guide wire re-crossing point in the imaging more frequently with more usage of the double lumen catheter. Hence, more Japanese experts focus on optimal SB dilation with clearance of jailed struts under the practical guidance of the imaging, which results in more SB dilation and less SB stenting. Since most of the previous reports were based on angio-guided procedure, suboptimal KBI which occupied more than 50% of the cases in the 3-dimentional OCT observation [36] has a potential risk of adverse cardiac events. Physiology-guided SB treatment is a smart strategy to reduce the complicated procedure and the stent failure induced by SB dilation which are hardly detected in the angiography. Imaging-guidance with high resolution, 3-dimentional OCT/OFDI mainly investigated in Japan, has a great potential to correct stent failure and lead to optimal procedure. Further study is warranted for its impact on long-term clinical outcome.

### Role of rotablation in bifurcation PCI

JBC experts performed more rotablation with a greater number of the burr under complete imaging-guidance. Since the calcification in the bifurcation has risks of stent underexpansion, carina shift, and SB occlusion [37], adequate ablation with rotablator is required. The OCT/OFDI can clearly illustrate the border of calcification and indicate residual thickness after rotablation accurately. Calcium thickness < 500–700 μm allows a scoring balloon to make crackles for adequate stent expansion [38, 39], which is the target index during the rotablation. Since the IVUS which Korean experts mainly use is inferior to the OCT/OFDI in the illustration of calcification, aggressive rotablation is unlikely to be promoted.

## Study limitations

The present study had some limitations. (1) Since this survey was performed on the selected experts of KBC and JBC, their therapeutic concept might be more specialized compared to nationwide average strategy. (2) Although there are significant statistical differences between KBC and JBC experts in several points, the small sample size might include some bias.

## Conclusion

JBC experts perform imaging-guidance more strictly at pre- and post-PCI as well as after MV stenting for the optimization of the bifurcation PCI. Moreover, they use rotablator more aggressively under OCT/OFDI guidance, whereas KBC experts mostly use FFR/iFR measurement and POT/re-POT which are currently recommended. JBC experts perform less 2-stenting instead of more performance of SB dilation after cross-over stenting. The most popular 2-stenting is different, that is, culotte stenting in JBC and crush stenting in KBC experts. Interventions for coronary bifurcation vary among the countries due to differences in social background and whether the basic strategy is determined based on the findings of angiography, imaging, or physiology.
